# Extracellular Soluble Membranes from Retinal Pigment Epithelial Cells Mediate Apoptosis in Macrophages

**DOI:** 10.3390/cells10051193

**Published:** 2021-05-13

**Authors:** Nayan Sanjiv, Pawarissara Osathanugrah, Emma Fraser, Tat Fong Ng, Andrew W. Taylor

**Affiliations:** Department of Ophthalmology, Boston University School of Medicine, Boston, MA 02118, USA; nsanjiv@bu.edu (N.S.); paw17@bu.edu (P.O.); esfraser@bu.edu (E.F.); tatfong@bu.edu (T.F.N.)

**Keywords:** exosomes, ocular immune privilege, alpha-melanocyte-stimulating hormone, apoptosis, immune regulation, membrane FasL

## Abstract

A central characterization of retinal immunobiology is the prevention of proinflammatory activity by macrophages. The retinal pigment epithelial cells (RPEs) are a major source of soluble anti-inflammatory factors. This includes a soluble factor that induces macrophage apoptosis when the activity of the immunomodulating neuropeptide alpha-melanocyte-stimulating hormone (α-MSH) is neutralized. In this manuscript, isolated extracellular soluble membranes (ESMs) from primary RPE were assayed to see if they could be the soluble mediator of apoptosis. Our results demonstrated that RPE ESMs mediated the induction of macrophage apoptosis that was suppressed by α-MSH. In contrast, the RPE line ARPE-19, cultured under conditions that induce similar anti-inflammatory activity to primary RPEs, did not activate apoptosis in the macrophages. Moreover, only the ESMs from primary RPE cultures, and not those from the ARPE-19 cell cultures, expressed mFasL. The results demonstrate that RPE ESMs are a soluble mediator of apoptosis and that this may be a mechanism by which the RPEs select for the survival of α-MSH-induced suppressor cells.

## 1. Introduction

The retinal pigment epithelial cells (RPEs) contribute to the ocular immune privilege by expressing soluble factors [1,2,3,4]. These factors regulate immune cell activity to preserve vision and protect the retina from the deleterious effects of inflammation [5]. The RPEs produce immune-regulating neuropeptides such as alpha-melanocyte-stimulating hormone (α-MSH) and neuropeptide Y (NPY) while expressing membrane-bound immune checkpoint molecules FasL, TRAIL, CTLA-2, and PD-L1 [3,4,6,7,8,9,10].

Using in situ cultures of RPE eyecups that maintain the integrity of the RPE monolayer in culture has demonstrated that the soluble immune-regulators in the conditioned media suppress the proinflammatory activity of macrophages, inhibit phagolysosome activation within phagocytes, and promote antigen-presenting cells to activate regulatory T cells [1,2,3,4,11]. When the neuropeptide α-MSH is depleted from the soluble factors generated by the RPE eyecups, the conditioned media induce apoptosis in macrophages [4,11]. A similar soluble apoptotic signal to macrophages is seen released by placental syncytiotrophoblasts of the immune-privileged maternal–fetal interface [12]. The soluble factor is extracellular vesicles (EVs) expressing membrane FasL (mFasL) and TRAIL. These EVs play a part in the required immune regulation to ensure fetal development and live birth.

There are several reports describing a potential role for RPE EVs in regulating immune cell activity [13,14,15,16]. While most of the analyses of RPE EVs are on EVs released by cultured RPE line ARPE-19 cells, they show that ARPE-19 EVs contain proteins that influence the activity of immune cells. It has been demonstrated that the RPE line ARPE-19 releases EVs with the potential to affect cellular functions including cell survival, metabolism, and immune suppression [13,14]. The ARPE-19 EVs suppress human T-cell proliferation and induce regulatory activity in human monocytes [14]. The histological analysis of RPE EVs stained for EV markers CD63 and CD81 shows EVs released by the RPEs, and on the basal side, the EVs can contain proteins associated with drusen formation in age-related macular degeneration [16]. RPE-derived EVs are also found in the conditioned media of human RPE eyecups [15]. However, the presence of active blebbing of the RPEs makes it possible for other soluble membranes, which express similar markers to the EVs with the surface proteins of RPEs, to be mixed within the isolated EVs [17]. Therefore, we isolated the extracellular soluble membranes (ESMs) released by RPEs in culture to see if they induce apoptosis in macrophages.

## 2. Materials and Methods

### 2.1. Cells

A monocytic leukemia cell line RAW264.7 (ATCC, Manassas, VA) was cultured in DMEM-F12 (Lonza, Walkersville, MD, USA) with 10% FBS (Lonza). The cells were passed twice a week and kept in culture and passed for no more than 3 months. Cells collected at passage were used for the experiments. The ARPE-19 cells were cultured in a manner that induces the cells to expresses immunoregulatory activity [18]. The ARPE-19 cells were collected from 2/3 confluent cultures and were replated into cultures in 10% FBS-DMEM-F12 (Lonza) and grown until confluent. The media were changed to SFM and the cultures were incubated for 24 h. The conditioned media (CM) were collected, cleared by centrifugation, and further processed to collect EVs.

Resting peritoneal macrophages were obtained from a peritoneal lavage of naive mice in 0.01 M PBS (Lonza). All experimental animal procedures were approved by the Boston University Institutional Animal Care and Use Committee in accordance with the NIH guide for the care and use of animals in research. Any red blood cells in the lavage were lysed using red blood cell lysing buffer (Sigma-Aldrich Corp, St. Louis, MO.) for 5 min on ice and centrifuged at 400× *g* for 5 min, and the cells were resuspended in RPMI 1640 with 10% FBS (Lonza). The cells were plated and incubated for 1 h. The media were replaced with serum-free media (SFM) made of RPMI 1640 supplemented with 10 mM HEPES, 1 mM sodium pyruvate, and nonessential amino acids (Lonza), along with 0.2% ITS+1, 0.1% BSA, and 10 µg/mL gentamycin (Sigma-Aldrich Corp, St. Louis, MO, USA) before treatment and assay.

### 2.2. Experimental Autoimmune Uveitis (EAU)

The C57BL/6 mice (Jackson Laboratories, Bar Harbor, ME, USA) were housed in the Boston University Animal Science Center. All experimental animal procedures were approved by the Boston University Institutional Animal Care and Use Committee in accordance with the NIH guide for the care and use of animals in research. EAU was induced by immunizing the mice as previously described [19]. The mice were immunized with an emulsion of complete Freund’s adjuvant of 5 mg/mL desiccated *Mycobacterium tuberculosis* (Difco Laboratories, Detroit, MI, USA) and 2 mg/mL interphotoreceptor retinoid-binding protein peptide (amino acids 1–20, Genscript, Piscataway, NJ. USA). The mice were injected i.p. with 0.3 µg pertussis toxin (Sigma-Aldrich Corp) and given a second injection of pertussis toxin two days later. The EAU was clinically scored every 3 to 4 days by microscopy fundus exam using the standard 5-point clinical score. When the chronic stage of EAU was reached, which is a sustained EAU score of 3 for all the mice, the mice were euthanized, and the eyes were enucleated for EAU RPE eyecups. Eyes were also collected from naive mice for RPE eyecups to use as normal controls.

The RPE eyecups were made as previously reported [1,2,3,4]. The enucleated eye was dissected in ice-cold PBS. A circumferential cut was made just below the ciliary body, and the anterior portion of the eye (cornea, iris, ciliary body, and lens) was removed, followed by the removal of the neural retina. The eyecups of RPE monolayer and underlying choroid and sclera were placed into a well of a 96-well round-bottom tissue culture plate (Corning, Corning, NY, USA) containing 200 µL SFM. The cultures were incubated for 24 h at 37 °C in 5% CO_2_. The conditioned media were collected and centrifuged, and the supernatant was collected and used as RPE-eyecup CM.

### 2.3. Extracellular Membrane Isolation

The ESMs were isolated from RPE-eyecup CM and ARPE-19 CM using a precipitation method used to isolate exosomes [20]. To pull down membrane vesicles in size up to 300 nm in diameter, the CM was mixed with a half-volume equivalent of Total Exosomes Isolation kit solution (Invitrogen, ThermoFisher, Waltham, MA), followed with vigorous mixing for 1 min. The mixed solution was kept at 4 °C for 24 h and centrifuged at 10,000× *g* for 60 min. The supernatant was discarded, and the pellet was washed once and resuspended in SFM. The protein content of the isolated ESMs was assayed using a standard protein assay kit (Pierce BCA Protein Assay Kit, ThermoFisher, Waltham, MA, USA).

### 2.4. Caspase 3/7 Activity

Into each well of a 96-well culture plate was placed 3.2 × 10^3^ macrophages, and the cultures were incubated for two hours for macrophage adherence. The macrophages were washed twice with warm SFM, and into each well, an equal volume of EVs was added, and Apo-ONE Homogeneous Caspase 3/7 assay solution (Pomega, Madison, WI, USA) was added to the wells. The macrophages were treated with the equivalent of two RPE-eyecup ESMs (2 µg), or two ARPE-19 culture well ESMs (10 µg) were used. After 24 h of incubation, the fluorescence intensity was measured with a Synergy H1 (BioTek, Winoodski, VT, USA) plate-reader set for 488 nm excitation and 521 nm emission. To assay for the effects of α-MSH on the cell expression of caspase 3/7, the α-MSH neuropeptide (Bachem, Torrance, CA) was added to the macrophage cultures before the ESMs, and caspase 3/7 was assayed 24 h later.

### 2.5. TUNEL Assay 

To assay for apoptosis using the TUNEL assay, 4 × 10^4^ RAW264.7 cells or primary macrophages in SFM were seeded into the wells of a Nunc Lab-TekII 8-chamber slide (ThermoFisher). The slides were incubated at 37 °C in 5% CO_2_ for 18 h and washed twice with SFM. Into each well was added 200 µL of SFM containing 5 µg of either normal-eyecup or EAU-eyecup ESMs. The cultures were incubated for another 18 h, the supernatant was removed, and the cells were fixed in 4% paraformaldehyde (Electron Microscope Sciences, Hatfield, PA, USA) for 10 min. The cells were stained using ApopTag Red In Situ Apoptosis Detection Kit (Millipore Corp, Temecula, CA, USA), and counterstained with DAPI (MPI Biomedical, Irvine, CA). After staining, the cells were imaged by fluorescence microscopy using a FSX100 inverted fluorescent imaging microscope (Olympus, Center Valley, PA, USA).

### 2.6. Immunoblotting for FasL

An equivalent of one RPE-eyecup of ESMs (1 µg), or one ARPE-19 culture well of ESMs (5 µg) was run on a 4–12% NuPage gel (Invitrogen, ThermoFisher, Waltham, MA, USA), and the electrophoresed proteins were transferred to a nonfluorescent nylon filter. The filter paper was probed with a rabbit anti-FasL antibody (Santa-Cruz Biotechnology, SD, CA, USA), followed by a secondary anti-rabbit-IgG conjugated to DyLight 649 (Rockland, Limerick PA, USA). The stained filters were imaged in a BioRad (Hercules, CA, USA) VersaDoc imaging system using two-color fluorescence to detect RPE65 autofluorescence and immunostained FasL-bands. From the digital images, the band intensities were quantified using Quantity One software (BioRad).

### 2.7. Statistical Analysis

One-way Kruskal–Wallis ANOVA with Dunn’s multiple comparisons and unpaired, nonparametric Kolmogorov–Smirnov t-tests were done to find statistical differences of *p* ≤ 0.05 using Prism software v. 9.1.0 Mac OS. Each experiment was run at least 3 times independently, and the results are presented as mean ± SEM. 

## 3. Results

### 3.1. RPE ESMs Induce Apoptosis in RAW264.7 Macrophage Cells 

**Figure 1:**
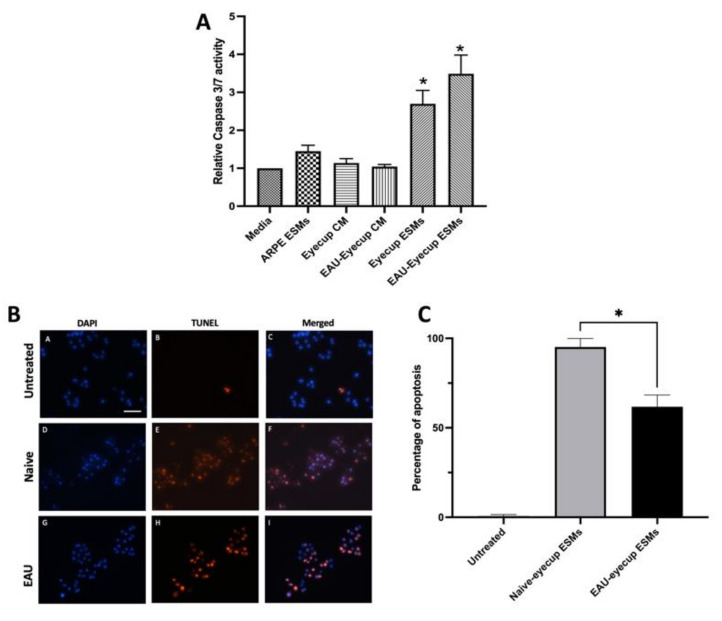
Induction of apoptosis in macrophages by RPE ESMs. The RAW264.7 macrophages were treated with whole conditioned media (CM) of naive or EAU eyecups (Eyecup CM or EAU-Eyecup CM), the ESMs from the conditioned media of naive or EAU eyecups (Eyecup ESMs or EAU-Eyecup ESMs), or the ESMs from confluent ARPE-19 cell cultures (ARPE ESMs) or were untreated (Media). (**A**) After 24 h, the cells were assayed for caspase 3/7 activity. Presented is the relative fluorescence (caspase 3/7 activity) to the background of untreated macrophages (Media) as mean ± SEM for 6 independent experiments for the EV-treated macrophages and 3 independent experiments for the conditioned-media-treated macrophages. Statistically significant (* *p* ≤ 0.001) levels of caspase 3/7 activity were seen only in the cultures of the macrophages treated with eyecup ESMs and EAU-eyecup ESMs in comparison with media alone. (**B**) Representative TUNEL-staining micrographs of RAW 264.7 macrophages treated with ESMs obtained from naive and EAU RPE eyecup for 24 h. Visual observations showed no TUNEL-positive cells in the untreated macrophage cultures (micrographs A–C), with most macrophages being TUNEL-positive after ESM treatment (micrographs D–I). Scale bar = 50µm. (**C**) The cells from the TUNEL assay were counted, and the percentage of TUNEL-positive cells over total DAPI-stained cells is presented as the mean ± SEM. The macrophages treated with naive ESMs had a significantly (* *p* ≤ 0.05) higher percentage of TUNEL-positive cells (95 ± 2%) than macrophages treated with EAU ESMs (62 ± 3%). The ESMs isolated from RPE eyecups (primary RPE cultures) induce apoptosis in the macrophage cell line.

It was previously reported that there is a soluble factor made by RPEs that induces apoptosis in macrophages [4,11]. To see if the ESMs from RPEs can induce apoptosis in macrophages, we treated the RAW264.7 macrophage cell model with RPE-derived ESMs and assayed for caspase 3/7 activity and DNA breaks by TUNEL staining. The ESMs were isolated from the conditioned media of RPE eyecups from naive and EAU mice. In addition, ESMs were isolated from confluent cultures of ARPE-19 cells [18]. The ESMs from the conditioned media of RPE eyecups from both naive and EAU eyes significantly activated caspase 3/7 activity (Figure 1A). In contrast, the ESMs from confluent ARPE-19 cells did not induce caspase 3/7 activity, nor did treating the macrophages with whole conditioned media (CM) from naive and EAU RPE-eyecup cultures. The cells were assayed for DNA fragmentation by TUNEL staining (Figure 1B,C). The ESMs from both naive and EAU RPE-eyecup cultures induced completion of apoptosis in RAW264.7 macrophages. The percentage of cells TUNEL-stained was significantly greater with the ESMs from naive RPE-eyecup cultures. These results showed that ESMs from the conditioned media of RPE eyecups induced apoptosis. While there was no difference in caspase 3/7 activation, the ESMs from naive RPE eyecups mediated a higher level of completed apoptosis in the macrophages than the ESMs from EAU RPE eyecups. In contrast, the ESMs from ARPE-19 cells did not induce apoptosis, which showed another significant difference from primary RPE cells.

### 3.2. The Effects of α-MSH on RPE-ESM-Induced Apoptosis

**Figure 2:**
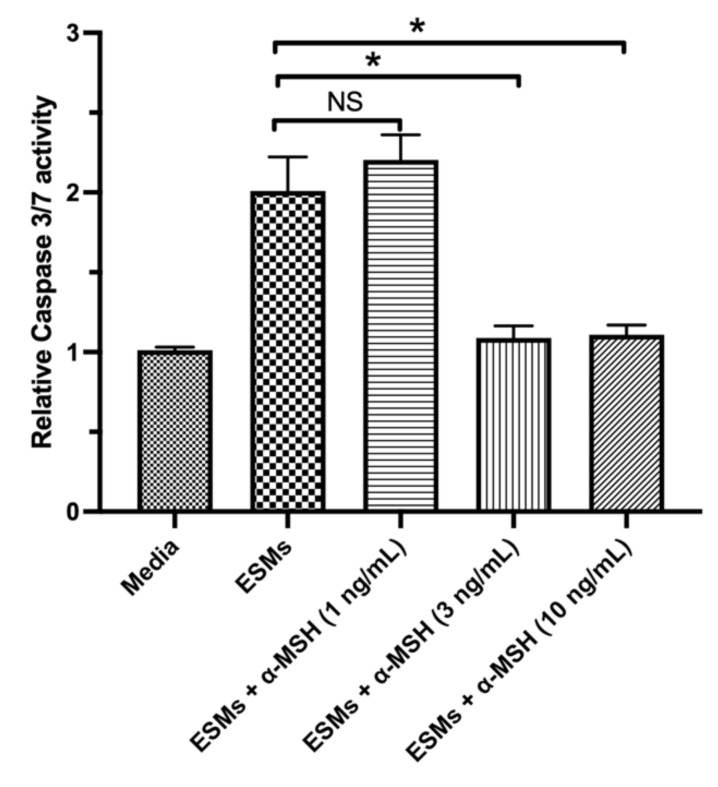
The effects of α-MSH on ESM-mediated apoptosis in macrophages. The RAW264.7 macrophages were treated with the eyecup ESMs with the addition of α-MSH (1, 3, or 10 ng/mL) (EV + α-MSH) or with no α-MSH (ESMs). In addition, cultures of macrophages in media (Media) were used as a background control. The cells were assayed after 24 h of incubation for caspase 3/7 activity. Presented is the relative fluorescence (caspase 3/7 activity) to the background media of untreated macrophages as mean ± SEM for 7 independent experiments. NS: not significant. Statistically significant (* *p* ≤ 0.001) suppression of caspase 3/7 activity was seen in the cultures of the eyecup EV-treated macrophages with 3 and 10 ng/mL of α-MSH in comparison with EV-treated macrophages with no α-MSH added (EV). Treating the macrophage cells with α-MSH suppressed the activation of caspase 3/7 induced by the RPE ESMs.

The conditioned media of naive RPE-eyecup cultures induced apoptosis in the macrophages only when the immunomodulatory neuropeptide α-MSH was removed from the conditioned media [4,11]. In addition, α-MSH provides survival signals to various cells of the retina and macrophages [21,22,23,24]. Therefore, the RAW264.7 macrophage cells were treated with α-MSH and then with the apoptosis-inducing ESMs from naive mouse RPE-eyecup cultures. It is reported that after 24 h of incubation there was 1–5 ng/mL of α-MSH in the RPE-eyecup conditioned media [4]. In a dose-dependent manner, α-MSH significantly suppressed the activation of caspase 3/7 induced by the ESMs from naive RPE-eyecup cultures to background levels (Figure 2). This suggests that the α-MSH produced by the RPE provides a survival signal to macrophages in the presence of apoptosis-inducing ESMs.

### 3.3. The Expression of Membrane FasL (mFasL) in RPE ESMs

**Figure 3:**
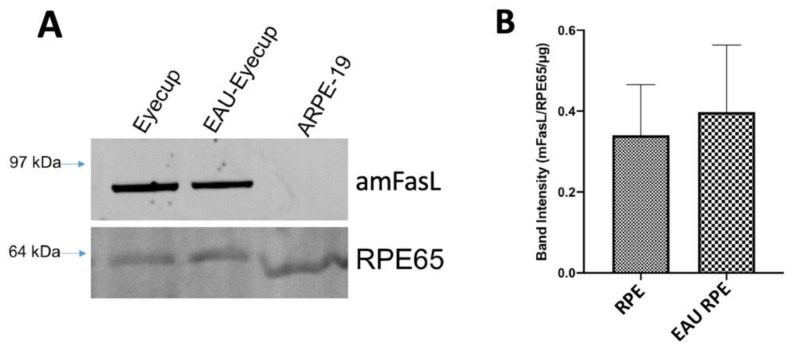
Expression of mFasL in RPE ESMs. (**A**) Immunoblot analysis for FasL was done on the isolated ESMs from RPE eyecups of naive and EAU eyes (Eyecup, EAU-Eyecup), and from the confluent cultures of ARPE-19 cells (ARPE-19). Bands that correspond with aggregated mFasL (amFasL) were detected in the ESMs from the RPE eyecups of healthy and EAU eyes and not from the ARPE-19 ESMs. Presented is a representative of 4 immunoblots. No FasL band was detected or measured in any EV from the culture supernatant of confluent ARPE-19 cells. (**B**) In all the ESMs, RPE65 was detected and used as a loading control along with the amount of protein loaded to measure the relative expression of the amFasL band intensity between the ESMs from naive (RPE) and EAU (EAU RPE) eyecups. Presented is the mean ± SEM of 4 independent experiments. There were no statistical differences between healthy and EAU EV expression of mFasL. The ESMs from RPE express mFasL.

One mechanism by which the ESMs could induce apoptosis in the macrophages could be through the expression of membrane FasL (mFasL), like what was seen in the immune-privileged maternal–fetal interface [25]. The ESMs were isolated from the conditioned media of the RPE eyecups and from cultures of confluent ARPE-19 cells. The ESMs’ protein cargo was assayed by immunoblotting for FasL. The ESMs from naive and EAU RPE-eyecup cultures had a highly detectible band that corresponds to aggregated mFasL (amFasL; Figure 3A and Supplementary Materials), the hexameric and functional form of mFasL [26]. The same amFasL is seen in ESMs from placental syncytiotrophoblasts [12]. Corresponding with the lack of caspase 3/7 activity by ESMs from confluent ARPE-19 cell cultures, there was no detectible FasL in the ARPE-19 ESMs (Figure 3A). When the immunoblots were imaged at 488 nm, a clear band for the autofluorescing RPE65 protein was seen from all the ESMs (Figure 3A). Since RPE65 is a constitutively produced, membrane-bound protein made only within and by RPEs, it provided a relative reference control that showed no difference in the expression of amFasL in the ESMs of naive and EAU RPE-eyecup conditioned media (Figure 3B). These results correspond with detecting caspase 3/7 activity and TUNEL staining induced by the ESMs from naive and EAU RPE-eyecup conditioned media.

### 3.4. RPE-ESM Induction of Apoptosis in Primary Macrophages

**Figure 4:**
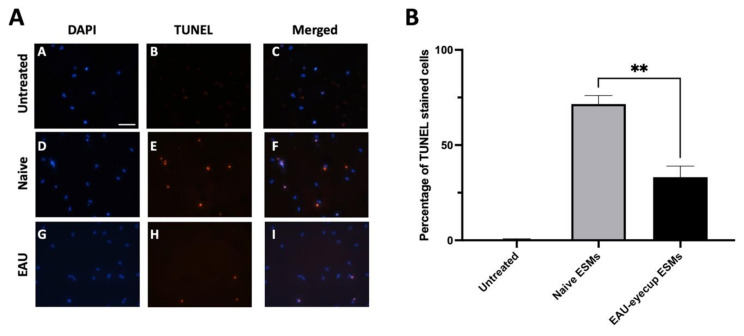
RPE ESMs induction of apoptosis in primary macrophages. The peritoneal macrophages from naive mice were treated with the ESMs from the conditioned media of naive RPE eyecups (Naive) or EAU RPE eyecups (EAU) or were untreated (Media). (**A**) The cultures were incubated for 24 h and stained by TUNEL assay. No TUNEL-positive cells were observed in the untreated cultures (micrographs A–C); TUNEL-positive cells were seen in the cultures of macrophages treated with ESMs from naive and EAU RPEs (micrographs D–I). Scale bar = 50µm. (**B**) The cells from the TUNEL assay were counted, and the percentage of TUNEL-positive cells is presented as the mean ± SEM. The macrophages treated with naive ESMs had a significantly (** *p* ≤ 0.01) higher percentage of TUNEL-positive cells (72 ± 4%) than macrophages treated with EAU ESMs (33 ± 6%). The ESMs isolated from RPE eyecups induce apoptosis in macrophages.

Previously, we showed that the conditioned media of RPE eyecups depleted of α-MSH induced apoptosis in primary macrophages as detected by TUNEL staining. To test whether the RPE ESMs induce apoptosis as suggested by the RAW cell experiments, the peritoneal macrophages from naive mice were cultured with isolated ESMs from naive or EAU RPE-eyecup cultures. The macrophages were assayed by TUNEL staining and showed a significant percentage of TUNEL-positive cells when treated with the ESMs from naive RPE-eyecup cultures (Figure 4). Moreover, as seen with the RAW cell cultures, there were significantly fewer TUNEL-stained macrophages treated with ESMs from EAU RPE-eyecup cultures. Therefore, the ESMs are a soluble factor from naive RPEs that can induce apoptosis in macrophages and potentially contribute to the mechanism of ocular immune privilege.

## 4. Discussion

Ocular immune privilege is the result of several adapted immune-regulating mechanisms constitutively expressed within the microenvironment of the eye [5]. A part of these mechanisms is derived from the RPEs, such as the immunomodulatory neuropeptide α-MSH and the immunosuppressive cytokine TGF-b. The results demonstrated that ESMs from RPEs have immunosuppressive properties in that they induce apoptosis in macrophages that are not suppressed or converted to regulatory cells by α-MSH. These results correspond with previous findings that apoptosis is induced in macrophages treated with the conditioned media of cultured RPE eyecups depleted of α-MSH [4,11]. In contrast, the ESMs from confluent cultures of ARPE-19 cells did not induce apoptosis in macrophages. This finding is not that much different from other reports of ARPE-19 cell EVs affecting macrophages in that the EVs can modulate immune activity [14]. In addition, the ARPE-19 EVs can regulate vascular growth and oxidative stress [13,27]. While the experiments were done based on protein concentration, which cannot be directly related to the number of exosomes or extracellular blebs in the conditioned media, there is a possibility that the differences seen could be due to the different types and amounts of extracellular vesicles required to mediate a response. Moreover, there is a possibility of an apical/basal polarization of ESMs by the RPEs that would have a polarity in cargo with different effects on cells under the RPEs and in the retina [16]. Our experiments did not sort out this possibility. While we used a method for efficient isolation of EVs, it is not possible to exclude the presence of RPE membrane blebs with similar sizes and markers [17,20]. Of the few published reports on the effects of primary RPE EVs and membrane blebs, our results suggest a potential for RPE ESMs contributing to the soluble molecules that regulate immune cell activity within the retina.

The expression of mFasL on the ESMs corresponded with the activation of caspase 3/7 and apoptosis within the macrophages. The potential role of FasL in ocular immune privilege has been known for some time [6,8]. The RPEs express high levels of FasL, which may be part of the blood–retinal barrier formed by the RPE monolayer in the back of the eye. The syncytiotrophoblasts of the placenta, another immune-privileged tissue site, release EVs that express the functional aggregate mFasL, which is dependent on membranes for formation [12,26]. These EVs induce extrinsic (FasL to Fas-mediated) apoptosis in T cells and monocytes, and this is considered an important mechanism of placental immunomodulation protecting the fetal tissues from lymphocytic attack triggered by fetal histoincompatibility. In the eye, apoptosis-mediating RPE ESMs would be part of a mechanism to prevent autoreactive and inflammatory immune activity. While we only assayed for FasL, there is the potential that other apoptosis-mediating molecules, such as TRAIL and proapoptotic miRNA, are carried by ESMs. The ESMs expressed an aggregated mFasL and induced caspase 3/7 activity; however, there was a significant reduction in macrophages treated with EAU-RPE ESMs that reached the final stages of apoptosis. The results suggest that even during inflammation, some mechanisms of ocular immune privilege remain expressed in the eye. This could be part of the self-resolving mechanisms of EAU in mice, and manufactured mFasL-expressing extracellular vesicles could be a therapeutic approach to uveitis.

**Figure 5:**
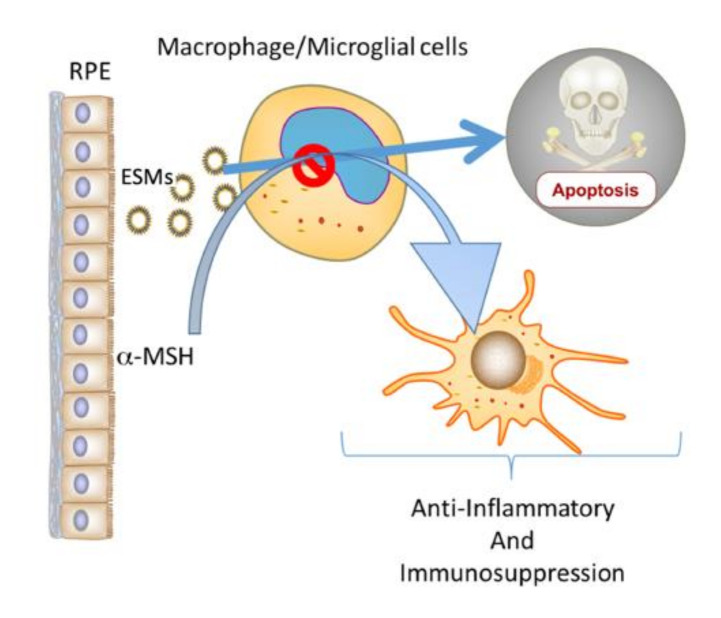
The potential role of ESMs and α-MSH produced by RPEs in ocular immune privilege. Within the immune-privileged eye, RPEs constitutively produce α-MSH and release ESMs. The results demonstrated that ESMs induce apoptosis in macrophages and that α-MSH suppresses the apoptotic signal. Previous publications have shown that α-MSH induces anti-inflammatory and suppressor activity in macrophages. Therefore, this combination of RPE ESMs and α-MSH could act as a selective mechanism that permits the survival of only macrophages and resident microglial cells that act to maintain the anti-inflammatory microenvironment of the eye.

The neuropeptide α-MSH is a potent modulator of immune cell activity [5,28,29,30,31,32,33]. It makes activated dendritic cells and macrophages suppress inflammation while activating Treg cells, and α-MSH converts effector T cells into Treg cells [22,29,30,32,33,34,35]. This effect of α-MSH on immune cells is a behavioral change in immune cells from mediating to suppressing inflammation and inducing tolerance. This places α-MSH with an important role in maintaining and mediating immune privilege [32]. The RPEs constitutively produce α-MSH that mediates RPE suppression of proinflammatory cytokines while promoting anti-inflammatory cytokine production by activated macrophages [3,4,11]. In addition, α-MSH can induce antiapoptotic activity in many cells, including macrophages [21,22,23,36,37]. The results demonstrated that the addition of α-MSH suppressed caspase 3 activation in the cultures of RPE-EV-treated macrophages. This implies that within the immune-privileged retina, macrophages and microglial cells would be rescued from the EV-induced apoptosis by α-MSH. However, the effects of α-MSH on these cells induce suppressive immune activity. Therefore, a mechanism of immune privilege could be a potential selection process in the retina for α-MSH-induced suppressor macrophages and microglial cells (Figure 5).

## 5. Conclusions

The results demonstrated that ESMs from cultured RPE eyecups can contribute to the soluble factors of RPE-mediated immunosuppression. The ESMs expressed mFasL and induced caspase 3 activity within macrophages. This apoptotic activity was suppressed by another soluble factor of RPEs, the neuropeptide α-MSH. Therefore, along with the other soluble immunoregulators released within the eye, RPE-released ESMs, which include exosomes, have the potential to contribute to the mechanisms of ocular immune privilege.

## Data Availability

The data presented in this study are available on request from the corresponding author.

## Supplementary Materials

Supplementary materials are available online at https://www.mdpi.com/article/10.3390/cells10051193/s1.

## References

[1] Zamiri P., Masli S., Kitaichi N., Taylor A.W., Streilein J.W. (2005). Thrombospondin Plays a Vital Role in the Immune Privilege of the Eye. Investig. Opthalmology Vis. Sci..

[2] Zamiri P., Masli S., Streilein J.W., Taylor A.W. (2006). Pigment Epithelial Growth Factor Suppresses Inflammation by Modulating Macrophage Activation. Investig. Opthalmology Vis. Sci..

[3] Lau C.H., Taylor A.W. (2009). The immune privileged retina mediates an alternative activation of J774A.1 cells. Ocul. Immunol. Inflamm..

[4] Kawanaka N., Taylor A.W. (2011). Localized retinal neuropeptide regulation of macrophage and microglial cell functionality. J. Neuroimmunol..

[5] Taylor A.W., Ng T.F. (2018). Negative regulators that mediate ocular immune privilege. J. Leukoc. Biol..

[6] Griffith T.S., Brunner T., Fletcher S.M., Green D.R., Ferguson T.A. (1995). Fas Ligand-Induced Apoptosis as a Mechanism of Immune Privilege. Science.

[7] Hueber A., Aduckathil S., Kociok N., Welsandt G., Dinslage S., Kirchhof B., Esser P.J. (2002). Apoptosis-mediating receptor–ligand systems in human retinal pigment epithelial cells. Graefe’s Arch. Clin. Exp. Ophthalmol..

[8] Ferguson T.A., Griffith T.S. (2007). The Role of Fas Ligand and TNF-Related Apoptosis-Inducing Ligand (TRAIL) in the Ocular Immune Response. Chem. Immunol. Allergy.

[9] Sugita S., Horie S., Nakamura O., Futagami Y., Takase H., Keino H., Aburatani H., Katunuma N., Ishidoh K., Yamamoto Y. (2008). Retinal Pigment Epithelium-Derived CTLA-2α Induces TGFβ-Producing T Regulatory Cells. J. Immunol..

[10] Ke Y., Sun D., Jiang G., Kaplan H.J., Shao H. (2010). PD-L1(hi) retinal pigment epithelium (RPE) cells elicited by inflammatory cytokines induce regulatory activity in uveitogenic T cells. J. Leukoc. Biol..

[11] Wang E., Choe Y., Ng T.F., Taylor A.W. (2017). Retinal Pigment Epithelial Cells Suppress Phagolysosome Activation in Macrophages. Investig. Opthalmology Vis. Sci..

[12] Stenqvist A.-C., Nagaeva O., Baranov V., Mincheva-Nilsson L. (2013). Exosomes Secreted by Human Placenta Carry Functional Fas Ligand and TRAIL Molecules and Convey Apoptosis in Activated Immune Cells, Suggesting Exosome-Mediated Immune Privilege of the Fetus. J. Immunol..

[13] Biasutto L., Chiechi A., Couch R., Liotta L.A., Espina V. (2013). Retinal pigment epithelium (RPE) exosomes contain signaling phosphoproteins affected by oxidative stress. Exp. Cell Res..

[14] Knickelbein J.E., Liu B., Arakelyan A., Zicari S., Hannes S., Chen P., Li Z., Grivel J.-C., Chaigne-Delalande B., Sen H.N. (2016). Modulation of Immune Responses by Extracellular Vesicles From Retinal Pigment Epithelium. Investig. Opthalmol. Vis. Sci..

[15] Locke C.J., Congrove N.R., Dismuke W.M., Bowen T.J., Stamer W.D., McKay B.S. (2014). Controlled exosome release from the retinal pigment epithelium in situ. Exp. Eye Res..

[16] Wang A.L., Lukas T.J., Yuan M., Du N., Tso M.O., Neufeld A.H. (2009). Autophagy and Exosomes in the Aged Retinal Pigment Epithelium: Possible Relevance to Drusen Formation and Age-Related Macular Degeneration. PLoS ONE.

[17] Alcazar O., Hawkridge A.M., Collier T.S., Cousins S.W., Bhattacharya S.K., Muddiman D.C., Marin-Castano M.E. (2009). Proteomics Characterization of Cell Membrane Blebs in Human Retinal Pigment Epithelium Cells. Mol. Cell. Proteom..

[18] Taylor A.W., Dixit S., Yu J. (2015). Retinal Pigment Epithelial Cell Line Suppression of Phagolysosome Activation. Int. J. Ophthalmol. Eye Sci..

[19] Lee D.J., Preble J., Lee S., Foster C.S., Taylor A.W. (2016). MC5r and A2Ar Deficiencies During Experimental Autoimmune Uveitis Identifies Distinct T cell Polarization Programs and a Biphasic Regulatory Response. Sci. Rep..

[20] Patel G.K., Khan M.A., Zubair H., Srivastava S.K., Khushman M., Singh S., Singh A.P. (2019). Comparative analysis of exosome isolation methods using culture supernatant for optimum yield, purity and downstream applications. Sci. Rep..

[21] Cheng L.-B., Cheng L., Bi H.-E., Zhang Z.-Q., Yao J., Zhou X.-Z., Jiang Q. (2014). Alpha-melanocyte stimulating hormone protects retinal pigment epithelium cells from oxidative stress through activation of melanocortin 1 receptor–Akt–mTOR signaling. Biochem. Biophys. Res. Commun..

[22] Taylor A.W. (2013). Alpha-Melanocyte Stimulating Hormone (α-MSH) Is a Post-Caspase Suppressor of Apoptosis in RAW 264.7 Macrophages. PLoS ONE.

[23] Maisto R., Gesualdo C., Trotta M.C., Grieco P., Testa F., Simonelli F., Barcia J.M., D’Amico M., Di Filippo C., Rossi S. (2016). Melanocortin receptor agonists MCR 1-5 protect photoreceptors from high-glucose damage and restore antioxidant enzymes in primary retinal cell culture. J. Cell. Mol. Med..

[24] Naveh N. (2003). Melanocortins applied intravitreally delay retinal dystrophy in Royal College of Surgeons rats. Graefe’s Arch. Clin. Exp. Ophthalmol..

[25] Kaminski V.D.L., Ellwanger J.H., Chies J.A.B. (2019). Extracellular vesicles in host-pathogen interactions and immune regulation—Exosomes as emerging actors in the immunological theater of pregnancy. Heliyon.

[26] Holler N., Tardivel A., Kovacsovics-Bankowski M., Hertig S., Gaide O., Martinon F., Tinel A., Deperthes D., Calderara S., Schulthess T. (2003). Two Adjacent Trimeric Fas Ligands Are Required for Fas Signaling and Formation of a Death-Inducing Signaling Complex. Mol. Cell. Biol..

[27] Maisto R., Oltra M., Vidal-Gil L., Martínez-Gil N., Sancho-Pellúz J., Di Filippo C., Rossi S., D’amico M., Barcia J.M., Romero F.J. (2019). ARPE-19-derived VEGF-containing exosomes promote neovascularization in HUVEC: The role of the melanocortin receptor 5. Cell Cycle.

[28] Clemson C.M., Yost J., Taylor A.W. (2017). The Role of Alpha-MSH as a Modulator of Ocular Immunobiology Exemplifies Mechanistic Differences between Melanocortins and Steroids. Ocul. Immunol. Inflamm..

[29] Namba K., Kitaichi N., Nishida T., Taylor A.W. (2002). Induction of regulatory T cells by the immunomodulating cytokines al-pha-melanocyte-stimulating hormone and transforming growth factor-beta 2. J. Leukocyte Biol..

[30] Taylor A.W., Alard P., Yee D.G., Streilein J.W. (1997). Aqueous humor induces transforming growth factor-ß (TGF-ß)-producing regulatory T-cells. Curr. Eye Res..

[31] Taylor A.W., Lee D.J. (2011). The Alpha-Melanocyte Stimulating Hormone Induces Conversion of Effector T Cells into Treg Cells. J. Transplant..

[32] Taylor A.W., Streilein J.W., Cousins S.W. (1992). Identification of alpha-melanocyte stimulating hormone as a potential immunosuppressive factor in aqueous humor. Curr. Eye Res..

[33] Taylor A.W., Streilein J.W., Cousins S.W. (1994). Alpha-Melanocyte-Stimulating Hormone Suppresses Antigen-Stimulated T Cell Production of Gamma-lnterferon. Neuroimmunomodulation.

[34] Lee D.J., Taylor A.W. (2013). Both MC5r and A2Ar Are Required for Protective Regulatory Immunity in the Spleen of Post–Experimental Autoimmune Uveitis in Mice. J. Immunol..

[35] Lee D.J., Taylor A.W. (2011). Following EAU Recovery There Is an Associated MC5r-Dependent APC Induction of Regulatory Immunity in the Spleen. Investig. Opthalmology Vis. Sci..

[36] Cai S., Yang Q., Hou M., Han Q., Zhang H., Wang J., Qi C., Bo Q., Ru Y., Yang W. (2018). A-Melanocyte-Stimulating Hormone Protects Early Diabetic Retina from Blood-Retinal Barrier Breakdown and Vascular Leakage via MC4R. Cell. Physiol. Biochem..

[37] Zhang Y., Bo Q., Wu W., Xu C., Yu G., Ma S., Yang Q., Cao Y., Han Q., Ru Y. (2015). α-Melanocyte-stimulating hormone prevents glutamate excitotoxicity in developing chicken retina via MC4R-mediated down-regulation of microRNA-194. Sci. Rep..

