# Supplementary material for: Extracellular Soluble Membranes from Retinal Pigment Epithelial Cells Mediate Apoptosis in Macrophages
Source: Cells. 2021 May 13;10(5):1193. doi: 10.3390/cells10051193 (PMC8153131; doi:10.3390/cells10051193)
Supplement: Supplementary file 1 [file cells-10-01193-s001.zip › cells-1184347-supplementary.pdf]

Supplementary Materials

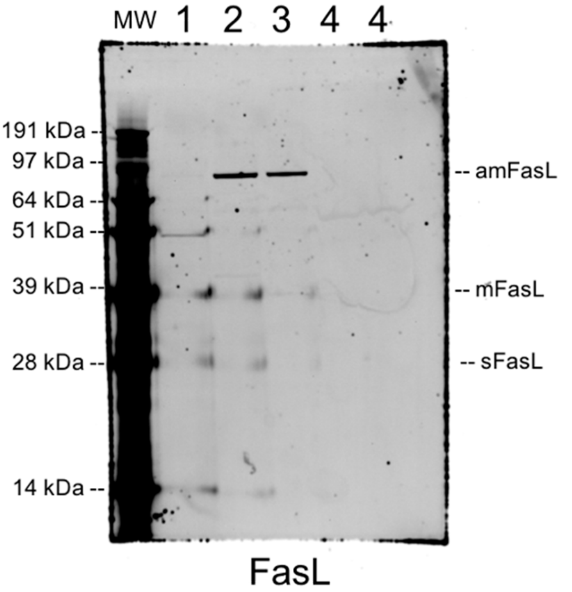

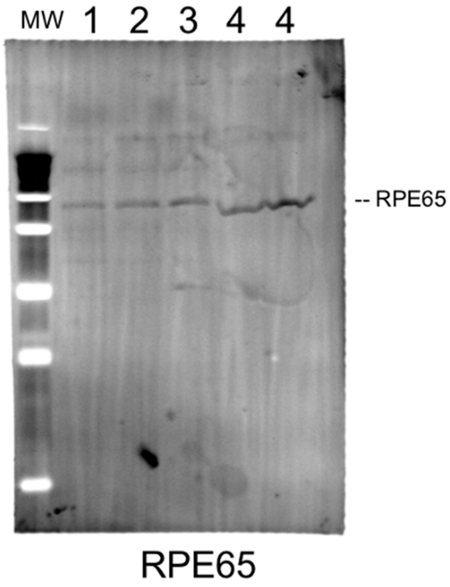

Supplemental Figure S1. Full images of the immunoblot presented in Fig. 3. The ESMs from RPE eyecups from eyes that were naive (lane 2) or with EAU (lane 3). ARPE-19 ESMs (lanes 4), and primary mouse-RPE cell lysate (lane 1) were assayed. MW = molecular weight markers.
